# Supplementary material for: An open-source framework for large-scale, flexible evaluation of biomedical text mining systems
Source: J Biomed Discov Collab. 2008 Jan 29;3:1. doi: 10.1186/1747-5333-3-1 (PMC2276192; doi:10.1186/1747-5333-3-1)
Supplement: Additional file 1 — Evaluation platform source code. The additional file contains the software evaluation framework discussed in this paper. The most current release can be downloaded from SourceForge.net [1]. See the accompanying README file for instructions on installation and use. Also included in the distribution is a collection of UIMA wrappers for some commonly used BioNLP tools and annotated corpora. [file 1747-5333-3-1-S1.zip › bionlp_uima_repository-1.0.1/docs/api/edu/uchsc/ccp/util/RunExecutable.html]

RunExecutable (Center for Computational Pharmacology Util/UIMA API)


|  |  |  |
| --- | --- | --- |
| **Overview** \| **Package** \| **Class** \| **Tree** \| **Deprecated** \| **Index** \| **Help** | |  |
| PREV CLASS   NEXT CLASS | **FRAMES**    **NO FRAMES**     **All Classes** |  |
| SUMMARY: NESTED \| FIELD \| CONSTR \| METHOD | DETAIL: FIELD \| CONSTR \| METHOD |  |


---


## edu.uchsc.ccp.util Class RunExecutable

```
java.lang.Object
  edu.uchsc.ccp.util.RunExecutable
```

---

``` public class RunExecutable extends Object ```

This class is a simple utility for calling command line executables.

**Author:**
:   William A. Baumgartner, Jr.

---

**Constructor Summary**

`RunExecutable()`


| **Method Summary** | |
| --- | --- |
| `static void` | `main(String[] args)`             This method used for testing purposes only. |
| `static void` | `run(String command, boolean showOutput)`             Run a command line executable. |

**Methods inherited from class java.lang.Object**

`clone, equals, finalize, getClass, hashCode, notify, notifyAll, toString, wait, wait, wait`

**Constructor Detail**

### RunExecutable

```
public RunExecutable()
```


**Method Detail**

### run

```
public static void run(String command,
                       boolean showOutput)
```

:   Run a command line executable.

    :   **Parameters:**: `command` - the command line function: `showOutput` - display output flag. Keep in mind that sometimes displaying output can be necessary to keep the Java process from moving on before the command line function completes.

---


### main

```
public static void main(String[] args)
```

:   This method used for testing purposes only.

    :   **Parameters:**: `args` - the command line arguments


---


|  |  |  |
| --- | --- | --- |
| **Overview** \| **Package** \| **Class** \| **Tree** \| **Deprecated** \| **Index** \| **Help** | |  |
| PREV CLASS   NEXT CLASS | **FRAMES**    **NO FRAMES**     **All Classes** |  |
| SUMMARY: NESTED \| FIELD \| CONSTR \| METHOD | DETAIL: FIELD \| CONSTR \| METHOD |  |


---
